# Phylogenetic analysis of ferlin genes reveals ancient eukaryotic origins

**DOI:** 10.1186/1471-2148-10-231

**Published:** 2010-07-29

**Authors:** Angela Lek, Monkol Lek, Kathryn N North, Sandra T Cooper

**Affiliations:** 1 Institute for Neuroscience and Muscle Research, The Children's Hospital at Westmead, Locked Bag 4001, Sydney, NSW 2145, Australia; 2 Discipline of Paediatrics and Child Health, Faculty of Medicine, University of Sydney, Sydney, Australia

## Abstract

**Background:**

The ferlin gene family possesses a rare and identifying feature consisting of multiple tandem C2 domains and a C-terminal transmembrane domain. Much currently remains unknown about the fundamental function of this gene family, however, mutations in its two most well-characterised members, dysferlin and otoferlin, have been implicated in human disease. The availability of genome sequences from a wide range of species makes it possible to explore the evolution of the ferlin family, providing contextual insight into characteristic features that define the ferlin gene family in its present form in humans.

**Results:**

Ferlin genes were detected from all species of representative phyla, with two ferlin subgroups partitioned within the ferlin phylogenetic tree based on the presence or absence of a DysF domain. Invertebrates generally possessed two ferlin genes (one with DysF and one without), with six ferlin genes in most vertebrates (three DysF, three non-DysF). Expansion of the ferlin gene family is evident between the divergence of lamprey (jawless vertebrates) and shark (cartilaginous fish). Common to almost all ferlins is an N-terminal C2-FerI-C2 sandwich, a FerB motif, and two C-terminal C2 domains (C2E and C2F) adjacent to the transmembrane domain. Preservation of these structural elements throughout eukaryotic evolution suggests a fundamental role of these motifs for ferlin function. In contrast, DysF, C2DE, and FerA are optional, giving rise to subtle differences in domain topologies of ferlin genes. Despite conservation of multiple C2 domains in all ferlins, the C-terminal C2 domains (C2E and C2F) displayed higher sequence conservation and greater conservation of putative calcium binding residues across paralogs and orthologs. Interestingly, the two most studied non-mammalian ferlins (Fer-1 and Misfire) in model organisms *C. elegans *and *D. melanogaster*, present as outgroups in the phylogenetic analysis, with results suggesting reproduction-related divergence and specialization of species-specific functions within their genus.

**Conclusions:**

Our phylogenetic studies provide evolutionary insight into the ferlin gene family. We highlight the existence of ferlin-like proteins throughout eukaryotic evolution, from unicellular phytoplankton and apicomplexan parasites, through to humans. We characterise the preservation of ferlin structural motifs, not only of C2 domains, but also the more poorly characterised ferlin-specific motifs representing the DysF, FerA and FerB domains. Our data suggest an ancient role of ferlin proteins, with lessons from vertebrate biology and human disease suggesting a role relating to vesicle fusion and plasma membrane specialization.

## Background

The ferlin family of genes in humans and most mammals is composed of six members, and possesses a distinct topology of tandem C2 domains (ranging from four to seven), with a single C-terminal transmembrane domain [1]. The ferlins were identified and named based on homology to a *Caenorhabditis elegans *spermatogenesis factor Fer-1 [2]. *C. elegans *Fer-1 mutants are infertile due to defective fusion of membranous organelles with the spermatozoan plasma membrane [3]. There are six mammalian ferlins (*Fer1L1-6*), with mutations in *FER1L1 *(dysferlin) and *FER1L2 *(otoferlin) linked to inherited diseases in humans [4,5]. Mutations in dysferlin underlie an autosomal recessive form of muscular dystrophy (limb girdle muscular dystrophy type 2B, LGMD2B and the allelic disorder Miyoshi myopathy) [4]. Mutations in oterferlin cause an autosomal recessive form of non-syndromic deafness (DFNB9) [5]. Dysferlin deficient mouse muscle fibers fail to perform calcium-dependent membrane resealing [6], a process thought to involve fusion and exocytosis [7,8] or endocytosis [9] of repair vesicles at sites of injury. Otoferlin null mice show a primary defect in calcium-mediated synaptic vesicle fusion and exocytosis at the synapse of cochlear inner hair cells [10]. *FER1L3 *(myoferlin) has not yet been linked to human disease, but studies of the myoferlin null mouse demonstrate impaired myoblast fusion and myofiber formation during development and regeneration [11]. Thus, emerging evidence points towards a common vesicular trafficking and fusion role for ferlin proteins, with unknown roles and tissue specificity for mammalian ferlins *FER1L4*, *FER1L5 *and *FER1L6*.

The occurrence of multiple tandem C2 domains that typify the ferlin family are rare. Only two other vertebrate gene families contain three or more C2 domains; the MCTP proteins (**m**ultiple **C**2 domain and **t**ransmembrane region **p**roteins) [12] and E-Syt (extended synaptotagmins) [13]. As an independent folding unit, C2 domains represent a functionally diverse and widely distributed calcium-binding motif. They are classically observed to function as calcium-dependent lipid binding modules [14], but some C2 domains have lost their calcium sensing ability and instead specialize in protein-protein interactions to regulate membrane trafficking and signal transduction [15]. Solved structures of C2 domains reveal a beta-sandwich fold consisting of eight antiparallel beta-strands connected by highly variable surface loops [16]. A key feature of calcium binding C2 domains is the conservation of calcium binding loops clustered on one end of the sandwich which are composed of negatively charged (usually aspartate) groups responsible for the coordination of multiple calcium ions [15]. In addition to influencing electrostatic potential to enhance phospholipid binding [17], the residue composition of this loop region also influences phospholipid selectivity, which allows for specificity of their target membranes [18].

Despite much effort dedicated to structurally and functionally characterize proteins with singly occurring C2 domains, not much is known regarding the purpose and function of multiple (greater than two) C2 domains. Should they be viewed as multiple instances of similarly functioning calcium binding units, maintained for efficiency or redundancy purposes? Or perhaps cross-talk and synergy between adjacent C2 domains gives rise to a more complex additive function beyond the classic role of C2 domains? Interestingly, sequence analysis and comparison of C2 domains within a single ferlin member reveal great diversity; where each domain is more similar to its corresponding counterpart in paralogs [19], indicating duplication from a common ancestral gene and evolution of specialized functions.

Another domain of interest subject to sequence analysis in this study is the DysF domain, which is present in only certain members of the ferlin family, and also in yeast peroxisomal proteins where its established function is to regulate peroxisome size and number [20]. This domain is of particular interest in understanding ferlin involvement in disease, as numerous disease-causing mutations in dysferlin have been mapped to both the inner and outer portions of the DysF domain. The DysF domain exists as an unusual nested repeat in ferlin proteins, where its function currently remains unestablished. The structure of the inner portion of the myoferlin DysF domain was recently solved, and shown to consist of two long antiparallel beta-strands. It has been suggested that preservation of function of both portions is likely, given that the insertion of the inner DysF domain occurs in an intervening loop region connecting the two beta-strands of the outer DysF domain, allowing both repeats to adopt the same fold without disruption to secondary structures [21]. In this study we present sequence analysis of the DysF domain of the ferlins, important for categorising and obtaining functional clues to this family of proteins.

The increasing availability of whole genome sequences and the annotation of genes from a wide range of phyla enables phylogenetic analysis of gene families to provide important contextual insight into their present day form and association with human disease. In this study, we report the phylogenetic analysis of the ferlin gene family, where we have retrieved ferlin genes from single-celled protists through to a range of metazoan species. We explored the diversity of ferlin domain topologies within this gene family, and examined the level of conservation of both C2 and non-C2 domain elements. The identification of both a DysF and non-Dysf ancestral ferlin in early metazoan species, and their expansion during vertebrate evolution, suggests a fundamental role associated with this ancient gene family that has specialized to include tissue-specific and isoform-specific functions. Sequence analysis within the ferlin protein family has thus far been limited to studies in higher vertebrates [19,22] with the exception of *C. elegans *[3]. In this manuscript we extend ferlin sequence analysis to include ferlin family members from eight metazoan phyla and three single-celled protists, some of which were derived from draft genomes.

## Methods

### Identification and annotation of unannotated ferlin genes

The ferlin genes for species within the Drosophila and Caenorhabditis genus and the unidentified mouse *Fer1L5 *was obtained by TBLASTN [23] searches against their respective databases. In most cases the ferlin gene was fully contained within a supercontig (or scaffold) and segment pairs were in close proximity to each other. The exons and exon boundaries were identified from the TBLASTN output as high scoring segment pairs or gaps within these segment pairs. In addition, exons were checked for correct ordering and strand. All hits were then manually analyzed for splice acceptor and donor sites to ensure the correct exon-intron boundaries. Finally, multiple sequence alignment using MAFFT was performed to ensure there were no gaps amongst orthologs from the Drosophila and Caenorhabditis genus.

### Domain classification

The locations of C2, DysFN, DysFC and transmembrane domains within ferlin genes were detected using SMART [24]. The location of FerI, FerA and FerB domains were detected using Pfam [25]. The program MAFFT was used to do multiple sequence alignments of DysFN, DysFC, FerA and FerB from various orthologs and paralogs [26]. CHROMA was used to highlight patterns in the resulting multiple sequence alignments [27]. Secondary structure prediction was performed using domains from human sequences as input into the Jnet secondary structure prediction server[28]. The resulting output was overlayed on the multiple sequence alignment generated for each domain.

The pair-wise sequence identity of C2 domains was determined using needle, a global alignment tool within EMBOSS [29]. Conservation of calcium-binding residues (aspartates and glutamates) were determined by alignment with C2A of Synaptotagmin 1 [Uniprot: P21579] for which key aspartates have been experimentally determined [30].

### Maximum likelihood trees

The program ClustalW2 [31] with default settings was used to perform multiple sequence alignment to use as input for PHYML. The phylogenetic trees based on protein sequences were generated using the maximum likelihood method employed by PHYML 3.0 [32] using a Le and Gascuel (LG) amino acid based model with estimated proportion of invariable sites and bootstrapping (100 replicates).

## Results

### Evolutionary relationship of ferlins in metazoans

**Figure 1:**
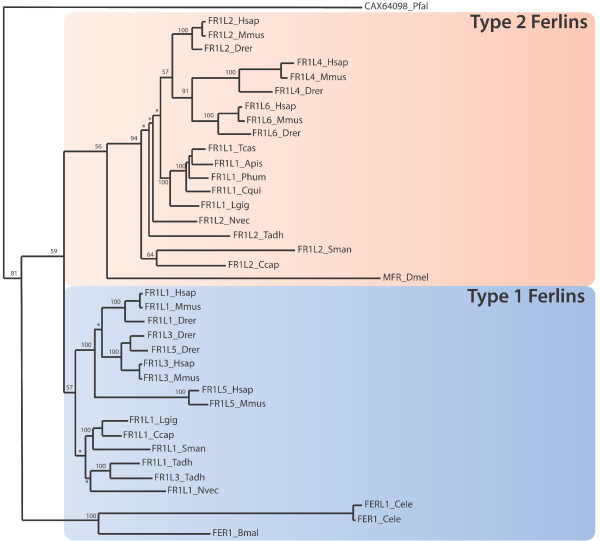
**Maximum likelihood tree of ferlin genes from selected phyla**. The putative ferlin from Pfal was used as an outgroup (CAX64098). Bootstrap values from 100 iterations are shown for major branches. Bootstrap values below 55 are indicated by an asterisk. DysF containing ferlins are partitioned into the bottom half of the tree (shaded blue box), while the non-DysF containing ferlins are partitioned into the top half of the tree (shaded orange box). FER1 and FERL1 of *C. elegans *form a separate sub-tree within the DysF subtree, while Misfire of *D. melanogaster *form an outgroup in the non-DysF subtree.

Much can be learnt about the evolution of genes and gene families from phylogenetic analysis. Given that mammalian ferlins are highly similar, little information can be inferred from their sequence comparisons. We therefore sought ferlin genes from selected phyla separated by larger evolutionary distances. Ferlin protein sequences from three protist and eight metazoan phyla (Table 1) were subjected to phylogenetic analysis (Figure 1). The SMART and Pfam database revealed no ferlin-specific domains amongst the prokaryotes. Results from maximum likelihood tree analysis reveals evolutionary partitioning of the ferlin protein family into two major subgroups (Figure 1); DysF-containing ferlins (Type I ferlins, blue shading) and non-DysF ferlins (Type II ferlins, orange shading). Metazoans typically have one or more ferlin of each subgroup.

**Table 1:** Ferlin protein sequences used in this study.

Species	Species Abbreviation	Common Name	Source	Accession
**Non-Metazoans**				

**Apicomplexa**				

Plasmodium falciparum	Pfal	Malaria parasite	Genbank	CAX64098
Cryptosporidium parvum	Cpar		Uniprot	Q5CS58, Q5CVS8

**Haptophyta**				

Emiliania huxleyi	Ehux	Phytoplankton	DOE JGI	Emihu1:464146

**Viridiplantae**				

Ostreococcus tauri	Otau		Uniprot	Q01FJ7
**Metazoans**				

**Cnidaria**				

Nematostella vectensis	Nvec	Sea anemone	Genbank	XP_001635151, XP_001628928

**Placozoa**				

Trichoplax adhaerens	Tadh		Genbank	EDV22852, EDV22851, EDV23443

**Annelida (Segmented worms)**				

Capitella sp. I	Ccap	Segmented worm	DOE JGI	Capca1:169002, Capca1:184805

**Mollusca**				

Lottia gigantea	Lgig	Gastropod snail	DOE JGI	Lotgi1:223003, Lotgi1:125050

**Platyhelminthes (Flat worms)**				

Schistosoma mansoni	Sman	Flat worm	UniProt	C4Q6T8, C4QI48

**Nematoda (Round worms)**				

Brugia malayi	Bmal	Round worm	UniProt	A8QEP1
Caenorhabditis elegans	Cele	Round worm	UniProt	Q17388, O01596

**Arthropoda**				

Pediculus humanus corporis	Phum	Body lice	Genbank	XP_002430476
Tribolium castaneum	Tcas	Beetle	Genbank	XP_968595
Acyrthosiphon pisum	Apis	Pea Aphid	Genbank	XP_001949781
Drosophila melanogaster	Dmel	Fruit Fly	Genbank	NP_001137919 (Misfire isoform F)
Culex pipiens quinquefasciatus	Cqui	Mosquito	Genbank	XP_001858804

**Chordata**				

Strongylocentrotus purpuratus	Spur	Sea urchin	Genbank	XP_001197550, XP_001194793, XP_001199804
Branchiostoma floridae	Bflo	Lancelet	UniProt	*C3Y3S6, C3XZR5, C3YBJ8*
Petromyzon marinus	Pmar	Lamprey	WUGSC	*Contig10491, Contig10569*
Callorhinchus milii	Cmil	Shark	A*STAR	*AAVX01058092, AAVX01535036, AAVX01036594, AAVX01262173, AAVX01071336*
Danio rerio	Drer	Zebra fish	Genbank/Uniprot	XP_689416, Q5SPC5, XP_687465, XP_001922278, XP_001923096, XP_001919573
Mus musculus	Mmus	Mouse	Genbank/Uniprot	Q9ESD7, Q9ESF1, Q69ZN7, A3KGK3.3, *, XP_982409
Homo sapiens	Hsap	Human	UniProt	O75923, Q9HC10, Q9NZM1, A9Z1Z3, A0AVI2, Q2WGJ9

**Key: **DOE JGI = Department of Energy Joint Genome Institute, A*STAR IMCB = Institute of Molecular and Cell Biology, WUGSC = Washington University Genome Sequencing Centre. * = Mmus FER1L5 was reconstructed using TBLASTN as described in methods. Sequence accession in italics indicate partial ferlin sequences.

Invertebrates generally have two ferlin genes (Nematoda and Arthropoda being exceptions, see below), one belonging in the non-DysF subgroup, and the other to the DysF subgroup, forming branching patterns similar to their evolutionary distance (i.e Tadh, Lgig, Ccap, Sman, Nvec; see Table 1). Two Dysf-containing ferlin sequences were evident in the invertebrate placozoan *Trichoplax adhaerens *(Tadh Fer1L1 and Fer1L3), which most likely result from a tandem duplication event (in scaffold 8), resulting in their close branching within the DysF subgroup.

**Figure 2:**
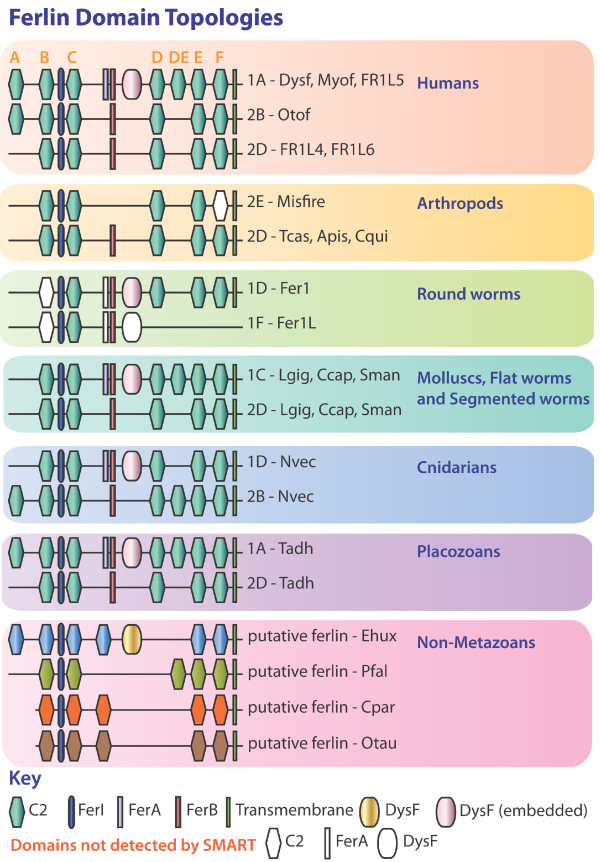
**Ferlin domain topologies**. The different variations in domain topologies observed for DysF containing ferlins (Type 1) and non-DysF containing ferlins (Type 2) are labelled A-F as follows - **A: **ferlin topology containing all seven C2 domains; **B: **(-)C2DE; **C: **(-)C2A; **D: **(-)C2A, (-)C2DE; **E: **(-)C2A, (-)C2DE, (-)FerB; **F: **(-)C2A, (-)C2 D, (-)C2E, (-)C2DE,(-)C2F. Domain classifications are an amalgamation of SMART (C2, DysF and transmembrane) and PFAM (FerA, FerB and FerI) results. The different C2 domains are labelled A-F according to their position from N-term to C-term. Domains colored in white are below threshold detection of SMART.

Nematoda and Arthropoda were noted exceptions to DysF-containing and non-DysF subgrouping. Nematodes (round worms) possess *only *DysF-containing ferlins (see also Figure 2). Phylogenetic analysis of ferlin sequences from two nematode species, *C. elegans *(Cele) and *Brugia malayi *(Bmal) form their own subgroup, clustering to neither Fer1L1/3/5 nor Fer1L2/4/6 subgroups, suggesting sequence divergence of ferlin proteins within the nematode phylum. *C. elegans *also has a closely related duplicate gene, *Fer1L1*, a truncated version of *Fer1 *lacking several C-terminal C2 domains and the transmembrane domain (see Figure 2).

Arthropoda possess *only *non-Dysf ferlins. Moreover, the Drosophila ferlin gene, Misfire (DmeI), is an outlier within the non-DysF subgroup, and does not cluster with ferlin sequences from other arthropods (Phum, Tcas, Apis, Cqui, see Table 1), despite having close evolutionary distance.

Vertebrates encode six ferlin proteins [19], with three in each Dysf subgroup. Vertebrate DysF-containing ferlins (Figure 1, blue box) then generally partition into Fer1L1-like (dysferlin), Fer1L3-like (myoferlin) and Fer1L5-like orthologs. Similarly, non-DysF ferlins generally cluster into orthologs of Fer1L2 (otoferlin), Fer1L4 and Fer1L6.

### Expansion of the ferlin gene family

To further explore the expansion of the ferlin family during vertebrate evolution, from two ferlin paralogs in invertebrates to six ferlin paralogs in vertebrates, we used draft genome sequences of the basal vertebrates lamprey (*Petromyzon marinus*, Pmar) and elephant shark (*Callorhinchus miliI*, Cmil)[33]. As the ferlin genes are quite large in comparison to sequences within contigs of draft genomes, it becomes impossible to find whole ferlin genes within a single scaffold. However, shorter stretches of conserved sequences encoding C2 domains (~100 amino acids) provide a plausible sequence length to detect in their entirety within a given scaffold. Analysis of sequence conservation amongst ferlin C2 domains revealed highest homology of C2E and C2F (discussed below in detail, see Table 2), and thus these two domains were separately employed as reference sequences to identify and extract ferlin genes from the lamprey and elephant shark draft genome sequences.

**Table 2:** C2 domain similarity compared to corresponding C2 domains from human dysferlin (Type 1) and otoferlin (Type 2).

Type	Ferlin	C2A	C2B	C2C	C2D	C2DE	C2E	C2F
1	FER1L1_Tadh	37.1	71.4	65	61	72	79	76.9

1	FER1L3_Tadh	47	69.4	62.7	59.1	49.3	80	51.4

1	FER1L1_Nvec		44.5	68.1	55.6		78	76.2

1	FER1L1_Ccap			68.7	60	75	75	80.8

1	FER1L1_Lgig		70.4	68.1	67	52.1	76	76.9

1	FER1L1_Sman		66.3	55.8	59.6		73	79.2

1	FER_Cele			34.6	41.8		66	49.3

1	FER1L1_Bmal			30.6	54.3	49.5	65.3	60.6

2	FER1L2_Tadh		73.3	64.9	66.1		87	72.5

2	FER1L2_Nvec	78.1	72.7	85.7	69.7	53.4	89	78.6

2	FER1L2_Ccap			56.4	64.2		71	78.6

2	FER1L2_Lgig		85.9	89.3	77.1		92	90.1

2	FER1L2_Sman		26.8	57.5	39.3		75	70.2

2	FER1L1_Phum		87.9	87.5	76.1		89	90.8

2	Misfire_Dmel		52.3	37.9	40		66	

**Key: **The C2DE domain from FER1L2_Nvec was compared to dysferlin C2DE since otoferlin does not contain this corresponding C2 domain. Blank spaces indicate C2 domains not detected by SMART.

**Figure 3:**
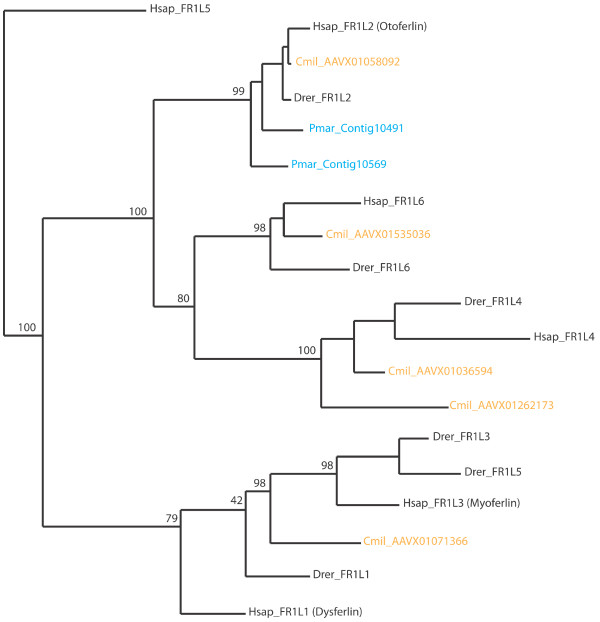
**Expansion of ferlin family occurred during vertebrate evolution**. Maximum likelihood tree constructed using protein sequences from ferlin C2F domains. Bootstrap values from 100 iterations are shown for major branches. Lamprey and elephant shark sequences are coloured in blue and orange, respectively. The inclusion of basal vertebrates lamprey (Pmar) and elephant shark (Cmil) demonstrate the expansion of the ferlin family during vertebrate evolution occurred after the divergence of jawless fish (lamprey) but before cartiligous fish (shark).

Ferlin tree topologies produced using C2E and C2F sequences, including those of Pmar and Cmil, produced a branching pattern (Figure 3) similar to those produced by the full-length ferlins (Figure 2), recapitulating DysF and non-Dysf subgrouping, and subtrees formed by paralogs. Our results indicate that there is enough information within C2F (and C2E) sequences to distinguish between ferlin paralogs, validating our approach in using this region for the retrieval of partial unannotated ferlin genes for producing maximum likelihood trees. Our results show that there are at least five ferlin paralogs in the shark (Cmil, low coverage of draft sequence may obscure a sixth ferlin gene) and at least two ferlin paralogs in the lamprey (Pmar). Therefore, our results suggest the expansion of the ferlin gene family from two to six ferlin genes occurred between the divergence of the jawless vertebrates (Pmar) and the cartilaginous fish (Cmil) during vertebrate evolution. Interestingly, the expansion of the ferlin gene family corresponds to whole genome duplication events thought to have occurred twice during vertebrate evolution, one near the divergence of jawless vertebrates and the other near the divergence of cartilaginous fish [34].

### Ferlin domain topologies

Figure 2 highlights the different domain topologies noted amongst the ferlin genes in this study. Genes which cluster within the DysF subgroup of Figure 1 are denoted as Type 1, and genes which cluster within the non-DysF subgroup are denoted as Type 2. Additionally, within each type there are subtle differences in domain topology giving rise to subtypes A-F (see Figure 2 legend). The major differences between the subtypes are the presence or absence of C2A (first N-terminal C2 domain), the FerA domain, or the C2DE domain (the C2 domain between C2 D and C2E).

All ferlin genes analysed possess multiple tandem C2 domains, with two highly conserved features: 1) An N-terminal FerI sequence sandwiched between two C2 domains (C2B-FerI-C2C motif); 2) Two C2 domains adjacent to a transmembrane sequence at the extreme C-terminus. All metazoan ferlins possessed these two features, with the exception of the putative truncated Fer1L of *C. elegans*, and reported truncated splice isoforms of Misfire [35]. These two features were also observed in putative ferlins of single-celled Apicomplexa such as the malaria parasite *Plasmodium falciparum *(Pfal).

Two outliers in terms of ferlin topology were truncated *C. elegans *Fer1L, and the Drosophila ferlin gene, Misfire. The unusual truncated topology of *C. elegans *Fer1L (see Figure 2) was also detected for *C. brenneri*, but not in three other analysed genomes within the Caenorhabditis genus (see Additional file 1). *C. elegans *Fer1L also has only a partial DysF domain, and it remains unclear whether Fer1L arose as a result of partial gene duplication, or whether a series of deletions followed a complete gene duplication of Fer-1. Misfire is distinct from all other arthropod ferlins, lacking the FerB domain and possessing a divergent C2F (end C-terminal C2 domain) that is below the C2 domain detection threshold of SMART.

### C2 domain conservation

Given that C2 domains represent the most abundant and distinctive feature of the ferlin gene family, we sought to establish the conservation of individual C2 domains throughout metazoan evolution (Table 2). A comparison of invertebrate ferlin C2 domains with human dysferlin and otoferlin revealed high sequence conservation in general, but particularly in the C-terminal C2 domains, C2E and C2F. In contrast, the N-terminal C2 domains have either been lost or have lower similarity. Human otoferlin and sea anemone ortholog Nvec FER1L2, are the only non-Dysf (Type 2) topology ferlins to maintain C2A, which is also absent in many invertebrate Dysf-containing (Type 1) topology ferlins. Interestingly, arthropod (Phum) and mollusc (Lgig) type 2 ferlins have very high sequence conservation of C2 domains when compared to otoferlin, suggesting ancestral functions/interactions may be preserved throughout metazoan evolution.

### Conservation of C2 domain calcium-binding residues

In classical calcium-sensitive C2 domains, there are five aspartates involved in the binding of calcium ions which results in electrostatic changes necessary for phospholipid binding [16]. In this study we have performed a multiple sequence alignment of dysferlin C2 domains against C2A of Synaptotagmin I to identify the corresponding calcium-binding residues by alignment (Table 3). Identification of calcium-binding residues in this case has allowed for the highly conservative D→E substitutions, as observed in some calcium-binding C2 domains [36]. C2E and C2F are shown to conserve classical calcium-binding residues across all six mammalian ferlin paralogs, and most invertebrate ferlin orthologs, with the exception of Fer1 of *C. elegans *and several apicomplexan parasites. Some ferlins show more C2 domains with potential calcium-binding, for instance, dysferlin (Fer1L1) shows sequence conservation of predicted calcium-binding aspartates in four C2 domains; C2C, C2 D, C2E and C2F. For ferlin C2 domains that lack the classical aspartate (or glutamate) residues, substitutions in the position of calcium-binding residues are often to a serine or asparagine, both of which are residue substitutions previously characterised to inactivate calcium-dependent phospholipid binding in C2A domain of synaptotagmins IV and XI [36].

**Table 3:** Conservation of five putative calcium coordinating residues in C2 domains.

	Dysferlin	Paralogs	Orthologs
C2A	No	N/A	N/A

C2B	No	N/A	N/A

C2C	Yes	No	No

C2D	Yes	No	Yes (1,2)

C2E	Yes	Yes	Yes (1)

C2F	Yes	Yes	Yes(1)

**Key: Yes **indicates that all five aspartates are conserved; **No **indicates all five could not be identified; **N/A **indicates these domains were not tested for aspartates as the corresponding dysferlin C2 domains have not conserved the five aspartates; **1 **indicates no conservation in Cele; **2 **indicates no conservation in Dmel. Residues considered as having potential for calcium coordination include aspartic and glutamic acid.

### Conservation of non-C2 domains

So far, no function or interaction has been attributed to the DysF, FerI, FerA or FerB domains in the ferlins. The highly conserved region preceding C2 D that is present in dysferlin, myoferlin and Fer1L5 has been termed DysF according to the SMART database, but is not annotated as a conserved/identifiable domain by the Pfam database. In contrast, the FerA, FerB and FerI regions are classified as domains by Pfam but not by SMART. Our analysis of metazoan phyla suggests all should be considered as domain units with potential function due to high conservation across orthologs and paralogs.

### FerA and FerB

**Figure 4:**
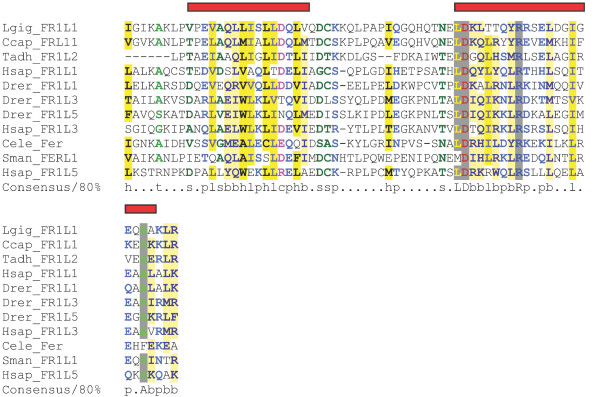
**FerA multiple sequence alignment**. FerA domain alignment of representative species from each phylum. The alignment was colored using CHROMA. Red bars indicate helix secondary structure prediction scoring above 8 using Jnet.

**Figure 5:**
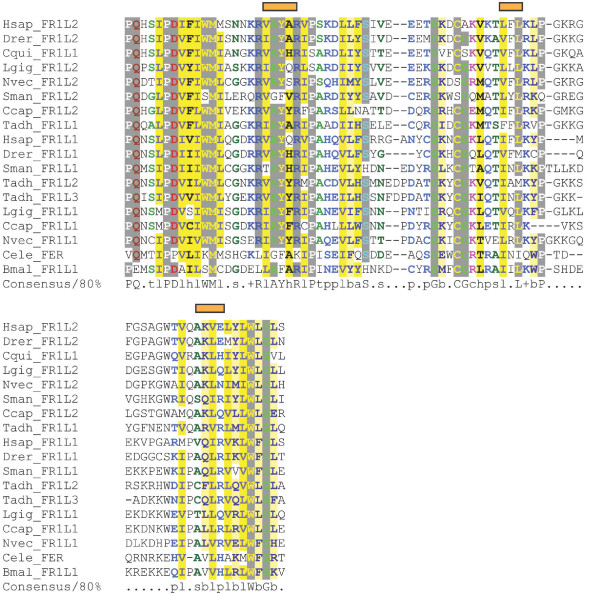
**FerB multiple sequence alignment**. FerB domain alignment of representative species from each phylum. The alignment was colored using CHROMA. Orange bars indicate beta strand secondary structure prediction scoring above 7 in Jnet.

The FerA domain of 66 amino acids in length and FerB domain of 76 amino acids in length occur midway between C2C and C2 D, and do not overlap with SMART's classification of the DysF domain. Both domains are unique to ferlin proteins, and show significant conservation of secondary structure elements as well as sequence conservation (Figures 4 and 5). Interestingly, FerB is conserved in all ferlins while FerA is only found in DysF containing ferlins (Type 1), suggesting FerA and DysF may have complementary or additive function.

### FerI and DysF

The FerI and DysF domains are of particular interest as both have been identified in ferlin-like proteins of unicellular eukaryotes. The FerI domain, but not the DysF domain, was discovered in ferlin-like proteins of protozoan Apicomplexan parasites Plasmodium (malaria), Theileria [Genbank:XP_765088] (East Coast fever), Babesia [Genbank:XP_001610088] (tick fever) and Toxoplasma [Genbank:XP_002364209] (toxoplasmosis) (data not shown), that account for significant worldwide mortality and morbidity amongst humans and livestock. Similar to the metazoan ferlins, the FerI domains in these Apicomplexan parasites are sandwiched closely between two C2 domains. This could suggest that the C2-FerI-C2 motif functions as a single entity and may have a fundamental function shared between metazoan and protozoan putative ferlins.

**Figure 6:**
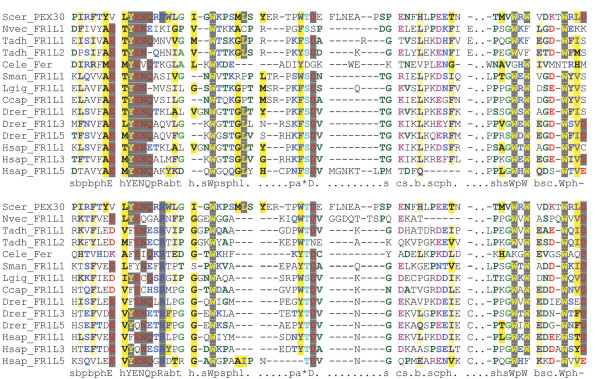
**DysFN multiple sequence alignment**. DysFN domain alignment of outer (top alignment) and inner (bottom alignment) from representative species from each phylum. The alignment was colored using CHROMA. The Pex30p DysFN sequence from *Saccharomyces cerevisiae *(Scer) is non-embedded with the same sequence used in the top and bottom alignment. The CHROMA consensus sequence shows sequence conservation despite embedding in metazoan sequences.

**Figure 7:**
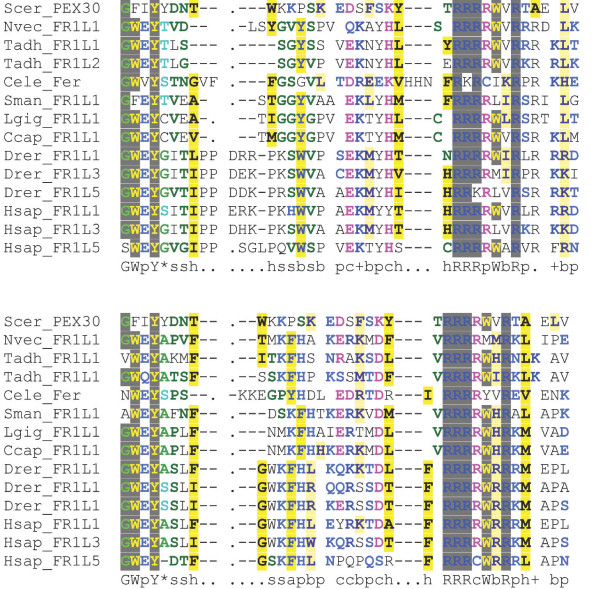
**DysFC multiple sequence alignment**. DysFC domain alignment of outer (top alignment) and inner (bottom alignment) from representative species from each phylum. The alignment was colored using CHROMA. The Pex30p DysFC sequence from *Saccharomyces cerevisiae *(Scer) is non-embedded with the same sequence used in the top and bottom alignment. The CHROMA consensus sequence shows sequence conservation despite embedding in metazoan sequences.

Figures 6 and 7 shows high sequence conservation of both inner and outer DysF domains, and confirms that its existence as a nested repeat has not caused major sequence divergence in either its inner or outer version, compared to the unembedded DysF in Pex30p. Interestingly, the DysF domain is also noted in one other human gene - an uncharacterised gene consisting of beta propeller repeats [Genbank:NP_056210] which contains two DysF domains, but not present in the embedded form, as is the case with the ferlins. A family of yeast peroxisomal proteins Pex30p, Pex31p and Pex32p has also been identified to contain a DysF domain, again as a non-embedded form [20]. Studies have specifically isolated the DysF domain in these genes as a regulator of normal peroxisome number and size. Thus the DysF domain is of ancient origins, and is uniquely present as an embedded repeat only in the ferlins (with the exception of unicellular protist Ehux with a single DysF domain that exists as an unembedded form).

## Discussion

Over recent years, advances in sequencing technology have led to the increasing pursuit of genome-wide sequencing of many species. The public availability of data via online databases has in turn enabled individuals to pursue phylogenetic research of their gene of interest to complement their laboratory studies. In this study we present a phylogenetic study, selecting representative vertebrate and invertebrate eukaryotic genomes to shed evolutionary insight into the characteristic features that define the ferlin gene family.

There are six ferlin genes identified in humans to date; three consisting of a DysF domain (Type 1), and three without (Type 2). However, our genomic analysis identified only two ferlins in invertebrates (one Type 1 and one Type 2), suggesting that the six mammalian ferlins originated from two ancestral ferlins of distinct subtypes. The need for metazoans to maintain ferlins of two different types suggests that DysF imparts a specific function, conserved throughout evolution. It is not clear whether the DysF domain was gained in an ancestral ferlin then maintained throughout evolution due to a selective advantage, or, whether the DysF domain was lost following a gene duplication event, also imparting a selective advantage. Arthropods and nematodes were observed as exceptions; a DysF-containing ferlin is not maintained in arthropods, while a non-DysF ferlin is not maintained in nematodes. Using highly conserved C2 domain sequences, we were able to identify and extract two ferlin paralogs in the lamprey (Cmil) and five in the shark (Pmar), narrowing down the likely expansion of the ferlin gene family between the divergence of the jawless vertebrates and the cartilaginous fish. Ferlin-like genes consisting of at least five C2 domains, a C-terminal transmembrane region, and a C2-FerI-C2 motif were also identified in five species of Apicomplexa parasites (Plasmodium, Cryptospiridium, Theileria, Babesia and Toxoplasma), and in unicellular phytoplankton (Ostreococcus), further supporting an ancient role of ferlin-like proteins in eukaryotic biology. Plasmodium and other apicomplexan parasites are characterised by a specialized apicoplast membrane, possess specialised secretory organelles (rhoptries) thought to be involved in events leading to host cell invasion, and form membrane vesicular structures termed 'parasitophorous vacuolar membrane' (PVM) in which the organism resides [37]. Given the large nature of the PVM (30-33um in surface area), the biological process which underlies its ability to form *de novo *in 10-20 seconds remains a curious area of research for many in the field [38]. With emerging roles for vertebrate ferlins in plasma membrane vesicle fusion [3,6,10], and the particular association of ferlins with cells possessing specialised plasma membrane networks such as skeletal and cardiac muscle [4], placenta [39], and sperm acrosome [40], a potential role for ferlins in specialist membrane networks of apicomplexan parasites provides an intriguing avenue for investigation.

Following the identification of the dysferlin gene in 1998, and the shared homology with Fer-1 of *C. elegans*, *Fer-1 *has since been thought of as the ancestral ferlin from which the human ferlins were derived. Our phylogenetic analysis of multiple invertebrate ferlins suggests that *Fer-1 *is not a typical ferlin gene. Despite some regions of homology, Fer-1 (and Drosophila Misfire) form outgroups in the ferlin phylogenetic tree (Figure 1). Fer-1 shows loss of conserved residues that define the DysF domain, while Misfire has lost the FerB domain present in all other metazoan ferlins. Maximum likelihood tree and intra-genus sequence comparison shows sequence divergence of Dmel and Cele from other species within their genus (Additional file 1 and 2), which is typical of rapidly evolving reproduction-related genes [41]. Misfire and Fer-1 may therefore present as exceptions, having lost and/or gained functions not shared across the ferlin family. Thus, we recommend caution when translating findings from studies of ferlin function in *Drosophila *and *C. elegans*, to that of ferlin-related diseases in humans, such as muscular dystrophy and non-syndromic deafness. Our results also highlight that species such as zebra fish (Drer) may represent a useful model organism, possessing the full complement of both DysF and non-DysF subgroups, and also technical utility for studies of muscle form and function.

In our sequence analysis of ferlin genes, we identified several conserved features amongst ferlin proteins; multiple tandem C2 domains (5-7), a single C-terminal transmembrane domain, a FerB domain, and a highly conserved N-terminal motif consisting of a FerI tightly sandwiched between two C2 domains. Slight variations in topologies include the incorporation of the optional C2A domain, the C2DE domain, and the FerA domain that appears to be concurrently present with the DysF domain.

Our analysis of the DysF domain shows it has maintained high sequence conservation. The presence of two DysF domains in a nested fashion is a unique and ancient feature of the ferlin family preserved from early diverging metazoans (Tadh, Nvec) through to humans. Despite resolution of the inner DysF domain structure of myoferlin, its function and purpose remains unknown [21]. Our analysis shows that nesting of the DysF has not caused sequence divergence in either the inner or outer DysF regions. Importantly, NMR structural studies have shown that the inner DysF inserts between important secondary structures of the outer DysF domain, thus allowing for both to fold into a similar structure. In addition, reported pathogenic mutations occurring within and between the inner and outer DysF domains of dysferlin suggests that the act of embedding has not disabled the functional capacity of this domain [21]. Interestingly, the DysF domain, although not in nested form, has also been reported to exist in yeast peroxisome proteins [20]. The function of the DysF domain in these proteins is to regulate peroxisome size and number. Therefore, given that dysferlin is localised to vesicles [6], the function of the DysF domain could analogously be hypothesized to regulate vesicle size and number.

In the species we have analyzed, the C-terminal C2 domains of the ferlins are shown to be more conserved than the N-terminal C2 domains. This suggests that the ferlin C-terminus is perhaps responsible for functions more fundamental, than specialized. In contrast, we observed more divergence within the N-terminus, whereby some species lack C2A, or possess a variation that is highly divergent. These data suggest the ferlin N-terminus could be responsible for functions that are more species specific, and/or ortholog specific, rather than unified across the ferlin family. Despite the lack of conservation of C2A and its absence in certain species, there is evidence to suggest that it remains functional in human ferlins. The solved structure of myoferlin C2A indicates that it is capable of folding into the characteristic C2 domain beta-sandwich, although consisting of more than the typical eight strands. In addition, an alternate splice isoform of C2A of unknown function, in which an alternate exon 1 is used in C2A, is reported to be expressed at significant levels in skeletal muscle and blood cells [42]. Furthermore, several missense mutations (Tryp52Arg, Val67Asp) lying within C2A are listed as probable muscular dystrophy causing changes. Dysferlin interaction with AHNAK, a protein implicated in membrane repair and maintenance has also been localised to C2A [43].

As seen in the ferlins, several C2 domains belonging to the synaptotagmin family also show degeneration of calcium-binding residues. In synaptotagmins, where structural data in addition to sequence data is available, these changes have been shown to confer ablation of calcium sensitivity [36]. Despite the caveat of lacking known structural data relating to the ferlins, sequence alignment with synaptotagmin C2 domains reveals high sequence and secondary structure conservation, with identifiable conservation of calcium-binding residues within many ferlin C2 domains. In our studies, four out of seven C2 domains in dysferlin show conservation of calcium-binding residues. A previous sequence analysis reports a similar result, although this study did not allow for the highly conservative D→E substitution [22], and therefore did not identify C2 D and C2E as potential calcium binding domains. Contrary to expectation, a study into the lipid binding specificities of dysferlin C2 domains reports calcium-*independent *phospholipid binding associated with the C2 domains presented here as most likely to possess calcium sensitivity [44]. It is however still plausible that these C2 domains possess a calcium-sensitive role, but one that is coupled to protein-protein interaction(s), as is the case with calcium-dependent t-SNARE binding in Synaptotagmin I [45]. In otoferlin, calcium-dependent binding to two t-SNARE proteins of the inner hair synaptic complex, syntaxin1A and SNAP-25 has been mapped to C2F [46], which in our study is shown to possess the full complement of calcium-binding residues. Another study also reports otoferlin C2A as capable of binding syntaxin1A, although in a manner that is calcium-independent, presumably due to the lack of calcium coordinating residues in the C2A domain [46].

The function and need for so many C2 domains in the ferlins remains a mystery. To draw further lessons from the synaptotagmin family, possible reasons could be attributed to preferential lipid recognition, differential calcium sensitivities, or an expanded repertoire of protein-protein interactions. When the crystal structure of the cytosolic portion of Synaptotagmin I was solved, it was discovered that C2A and C2B were facing in opposite directions [47]. This peculiar orientation was later attributed to the fact that both interact with two opposing membranes of different lipid composition, where C2A was shown to bind synaptic vesicle membranes, while C2B showed preference towards the PI(4,5)P2 rich plasma membrane. This idea of lipid selectivity and preferential binding has been corroborated to an extent, in studies which showed that dysferlin C2A could be distinguished from other C2 domains with its unique phosphoinositide binding ability not commonly seen in others [44].

The idea of synergy and co-operativity between tandem C2 domains is also another novel concept explored in synaptotagmins. Interestingly, studies of C2A and C2B in isolation show they display different properties than when the two are studied together in tandem. In isolation, C2A of Synaptotagmin I does not bind SNAREs, whereas C2B does not penetrate lipid membranes. However, when tethered to an adjacent C2 domain, C2A is able to bind SNAREs [48], whereas tethering C2B to C2A, even a non-functional version which fails to bind lipid or calcium, enables C2B binding and penetration of membranes. Experimental evidence reports the requirement for simultaneous neutralisation of calcium binding residues in both C2 domains of synaptotagmin to completely disrupt calcium triggered membrane and SNARE interaction, thus indicative of functional redundancy between adjacent C2 domains [48]. It remains to be established whether there is inter-domain co-operativity and/or functional redundancy shared between ferlin C2 domains.

In this study we have surveyed a range of domain topologies present amongst ferlin genes, highlighting highly preserved domain regions, as well as species-specific domain combinations. Together, this information provides valuable insight into the minimal components required to construct a basic ferlin-like gene. Research into mini-gene (truncated) therapeutic constructs for gene restoration is currently underway for other genes associated with muscular dystrophy such as dystrophin, whose size exceeds the 5kb AAV vector packaging limit [49]. We hypothesise that functional 'mini-ferlins' will require evolutionarily preserved C2-FerI-C2 (~274aa) and C2E-C2F-TM (~489aa) motifs, with (dysferlin) or without (otoferlin) the nested DysF repeat (~228aa embedded form).

In summary, we define ferlins as an ancient family of C2 domain-containing proteins that are likely to possess primordial functions in eukaryotic biology. Our studies demonstrate expansion of the ferlin family during vertebrate evolution; often a basis for functional specialization and tissue-specific expression. We provide useful contextual insight into evolutionary preservation of not only the C2 domains, but also the less studied DysF, FerI, FerA and FerB protein domains. Defining the roles of each of these domains is essential to delineate the biology of ferlins, with clinical relevance to inherited human disease (dysferlin and otoferlin), and perhaps more widely significant in future studies of apicomplexan parasitology.

## Conclusion

In this study we show that ferlins are an ancient family of genes common across protists and metazoans, but not in plants or fungi. Metazoans were shown to maintain distinct Type I (with a Dysf domain) and Type II (without a Dysf domain) ferlin lineages; with invertebrates generally possessing two ferlins, and vertebrates possessing six ferlins. Unexpectedly, ferlins from model organisms *C.elegans *(Fer-1) and *Drosophila *(misfire) presented as phylogenetic outgroups, suggesting reproduction-related divergence. All ferlins possess an N-terminal C2-FerI-C2 sandwich, a FerB motif, and two C-terminal C2 domains adjacent to an extreme C-terminal transmembrane domain. Preservation of these structural elements throughout eukaryotic evolution suggests a fundamental role of these motifs for ferlin function.

## Authors' contributions

SC and KN supervised the study. AL and ML conceived of the study and carried out the work. All authors read and approved the final manuscript.

## Supplementary Material

Additional file 1 **Maximum likelihood tree of ferlins from the Caenorhabditis genus**. Click here for file

Additional file 2 **Maximum likelihood tree of ferlins (Misfire) from the Drosophila genus**. Click here for file
