# Supplementary material for: Phylogenetic analysis of ferlin genes reveals ancient eukaryotic origins
Source: BMC Evol Biol. 2010 Jul 29;10:231. doi: 10.1186/1471-2148-10-231 (PMC2923515; doi:10.1186/1471-2148-10-231)
Supplement: Additional file 1 — Maximum likelihood tree of ferlins from the Caenorhabditis genus. [file 1471-2148-10-231-S1.PDF]

Additional File 1

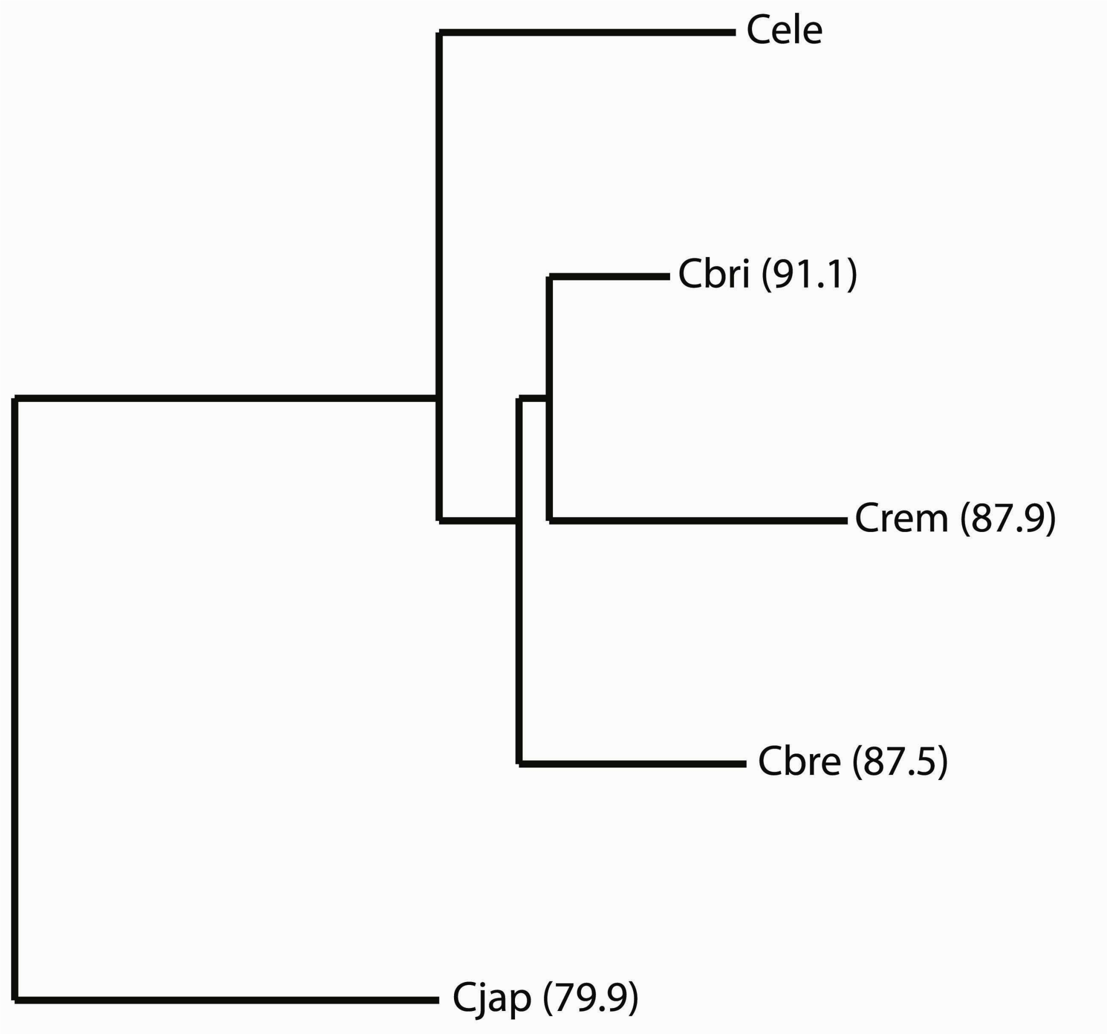

Maximum likelihood tree of ferlins from the Caenorhabditis genus. Numbers in brackets indicate pair-wise percent similarity compared to Fer-1 from Cele.

Caenorhabditis ferlin sequences.

Species	Species Abbreviation	Common Name	Source
**Caenorhabditis**			
Caenorhabditis elegans	Cele	Round worm	UCSC
Caenorhabditis brenneri	Cbre	Round worm	UCSC
Caenorhabditis briggsae	Cbri	Round worm	UCSC
Caenorhabditis remanei	Crem	Round worm	UCSC
Caenorhabditis japonica	Cjap	Round worm	UCSC

Key: Ferlin sequences of species from Caenorhabditis genus used to generate the above maximum likelihood tree. WUGSC = Washington University Genome Sequencing Centre.
