# Supplementary material for: Phylogenetic analysis of ferlin genes reveals ancient eukaryotic origins
Source: BMC Evol Biol. 2010 Jul 29;10:231. doi: 10.1186/1471-2148-10-231 (PMC2923515; doi:10.1186/1471-2148-10-231)
Supplement: Additional file 2 — Maximum likelihood tree of ferlins (Misfire) from the Drosophila genus. [file 1471-2148-10-231-S2.PDF]

Additional File 2

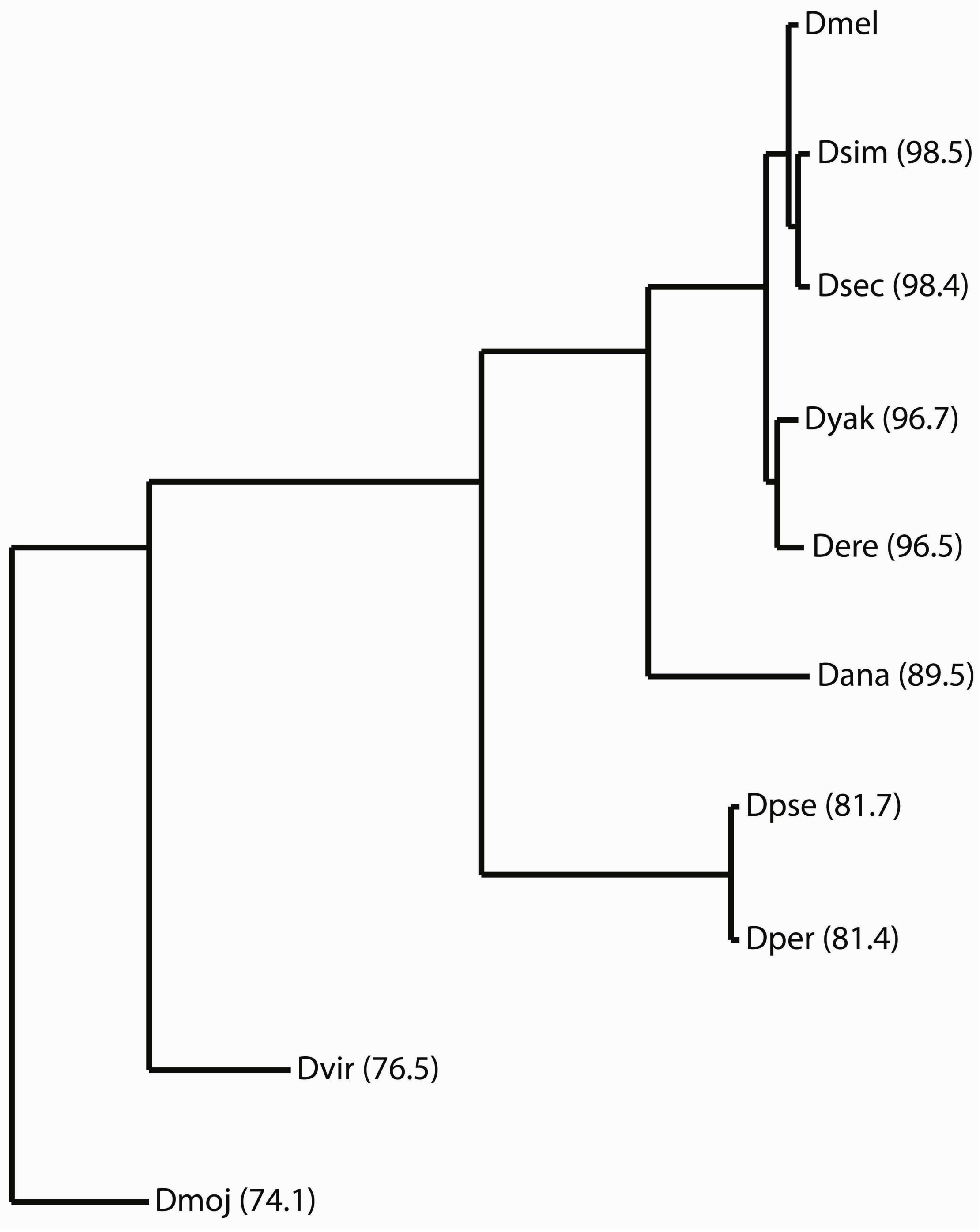

Maximum likelihood tree of ferlins (Misfire) from the Drosophila genus. Numbers in brackets indicate pair-wise percent similarity compared to Misfire from Dmel.

Drosophila ferlin sequences.

Species	Species Abbreviation	Common Name	Source
**Drosophila**			
Drosophila melanogaster	Dmel	Fruit fly	UCSC
Drosophila simulans	Dsim	Fruit fly	UCSC
Drosophila sechellia	Dsec	Fruit fly	UCSC
Drosophila yakuba	Dyak	Fruit fly	UCSC
Drosophila erecta	Dere	Fruit fly	UCSC
Drosophila ananassae	Dana	Fruit fly	UCSC
Drosophila pseudoobscura	Dpse	Fruit fly	UCSC
Drosophila persimilis	Dper	Fruit fly	UCSC
Drosophila virilis	Dvir	Fruit fly	UCSC
Drosophila mojavensis	Dmoj	Fruit fly	UCSC

Key: Ferlin sequences of species from Drosophila genus used to generate the above maximum likelihood tree. WUGSC = Washington University Genome Sequencing Centre.
